# Alterations in brain physiological pulsations in Parkinson's disease: a case-control study

**DOI:** 10.1016/j.ynirp.2026.100391

**Published:** 2026-07-30

**Authors:** Rodolphe Nenert, Corina Catiul, Pierre LeVan, Aya M. Miften, Jerzy P. Szaflarski, Amy W. Amara

**Affiliations:** a Department of Neurology, University of Alabama at Birmingham Heersink School of Medicine, Birmingham, AL, USA; b Department of Radiology, Cumming School of Medicine, University of Calgary, Calgary, Alberta, Canada; c Hotchkiss Brain Institute and Alberta Children's Hospital Research Institute, University of Calgary, Calgary, Alberta, Canada; d Department of Neurology, University of Colorado Anschutz Medical Campus, Aurora, CO, USA

**Keywords:** Parkinson's disease, Glymphatic clearance, Magnetic resonance encephalography

## Abstract

**Introduction:**

Brain physiological pulsations drive glymphatic clearance of neurotoxins from the brain. Neurodegenerative proteinopathies, including Parkinson's disease (PD), have altered glymphatic function. This study compares brain physiological pulsations important for glymphatic clearance in PD and healthy controls (HC).

**Methods:**

PD (N = 13) and HC (N = 20) participants were evaluated using magnetic resonance encephalography (MREG), an ultrafast, 3D spherical stack-of-spirals dynamic neuroimaging technique, to assess spectral power and optical flow in cardiovascular, respiratory, and low-frequency vasomotor bands as indirect proxies for glymphatic clearance. Group differences were analyzed with two-sample t-tests, controlling for age and sex.

**Results:**

PD participants demonstrated significantly reduced power in the cardiovascular frequency band and reduced optical flow magnitude in the low-frequency vasomotor band in regions important for visual and cognitive processing and sensorimotor function.

**Conclusion:**

PD is associated with alterations in physiological pulsations important for glymphatic flow. Glymphatic clearance may represent a potential therapeutic target for neurodegenerative diseases.

## Highlights

• Brain physiological pulsations drive glymphatic clearance.
• Magnetic resonance encephalography measures brain physiological pulsations.
• Cardiovascular frequency band power is reduced in PD compared to controls.
• Low-frequency vasomotor optical flow is reduced in PD compared to controls.
• PD alters physiological brain pulsations important for glymphatic flow.

## 1 Introduction

Parkinson's disease (PD) is a progressive neurodegenerative disease characterized by pathological accumulation of α-synuclein. The glymphatic system, which facilitates exchange of interstitial fluid and cerebrospinal fluid (CSF) along perivascular spaces through aquaporin 4 channels on astrocytic endfeet, is increasingly recognized as integral to clearance of neurotoxic proteins such as amyloid-β, tau, and α-synuclein from the brain (Iliff et al., 2012; Lopes et al., 2022). Thus, optimization of glymphatic clearance is a potential disease-modifying therapeutic target for proteinopathies such as PD (Lopes et al., 2022).

Glymphatic clearance is impaired in animal models of PD and other neurodegenerative disorders (Iliff et al., 2012; Lopes et al., 2022). Inhibition of glymphatic clearance in animal models leads to increased accumulation of α-synuclein in the brain, as well as motor dysfunction and brain atrophy (Lopes et al., 2025). The study of glymphatic clearance in humans is more challenging, and non-invasive imaging techniques are being explored to enhance our understanding of this complex system (Kiviniemi et al., 2016; Wood et al., 2024). While conventional neuroimaging techniques such as resting-state fMRI have been used to study brain pulsatility in PD (Shirzadi et al., 2018), their limited temporal resolution (∼0.5–3 s) prevents separation of distinct physiological frequency bands. Similarly, EEG captures neural electrical activity but does not measure the hemodynamic and fluid pulsations that drive glymphatic flow. A technique that addresses these limitations is magnetic resonance encephalography (MREG), a novel, ultrafast, 3D spherical stack-of-spirals dynamic imaging technique that detects physiological pulsations (Kiviniemi et al., 2016). These include cardiovascular, respiratory, and low-frequency vasomotor pulsations, which drive the convective flow of CSF through the glymphatic pathway and are fundamental to glymphatic function (Iliff et al., 2012; Kiviniemi et al., 2016; Rajna et al., 2019). Alterations in these pulsations have been observed in Alzheimer's disease, and our prior work has linked them to sleep architecture and cognitive performance in healthy older adults (Nenert et al., 2025; Rajna et al., 2021). The current study uses MREG to compare brain physiological pulsations between individuals with PD and healthy controls (HC), with the hypothesis that cardiovascular and low-frequency pulsations will be reduced in PD compared to HC, reflecting altered vascular pulsatility and perivascular fluid dynamics.

## 2 Methods

Thirteen Parkinson's disease (PD) patients (age: 62.3 ± 4.18 (mean ± SD); 9 M/4 F; Movement Disorders-Unified Parkinson's Disease Rating Scale (MDS-UPDRS): 44.31 ± 22.13; duration of disease: 5.54 ± 3.6 years; levodopa equivalent dose: 518.6 ± 292.1; side of symptom onset: left: 7 and right: 6) meeting UK Brain Bank clinical diagnostic criteria were scanned in their “ON” medication state and compared directly to twenty healthy adults (HC) (age: 67 ± 4.18; 8 M/12 F). A Welch's two-sample *t*-test showed no statistical difference between the groups in age (t ≈ 1.81, p = 0.089) or sex (χ^2^ ≈ 1.65, p = 0.20).

PD participants were enrolled from the University of Alabama at Birmingham (UAB) Movement Disorders Clinic and the UAB Sleep/Wake Disorders Center. Study staff and the PI introduced the trial during regular clinic visits, posted IRB-approved flyers in clinical and research areas, and attended community health fairs and local PD support groups. All participants provided written informed consent under UAB Institutional Review Board approval. All procedures were performed in compliance with relevant laws and institutional guidelines and were approved by the appropriate institutional committees. This work was performed under UAB IRB protocols IRB-300005901, which had initial approval on 9/22/20 and IRB-300008136, which had initial approval on 12/14/21.

Data were acquired on a Siemens 3T PRISMA using ultrafast magnetic resonance encephalography (MREG) sequence as previously described (TR = 100 ms, TE = 36 ms, flip angle = 25°, 3 mm isotropic voxels, 10 Hz sampling) (Nenert et al., 2025). Each participant underwent two 10-min resting-state runs with eyes open. The ultrafast MREG acquisition (100 ms per each whole brain volume) inherently minimizes inter-volume displacement, as the time between consecutive volumes is too brief for substantial motion to occur, reducing the likelihood of generating motion artifact that affects the scans. An anatomical 3D Magnetization Prepared Rapid Acquisition with Gradient Echo (MPRAGE) was acquired with TR = 1900 ms, TE = 2.49 ms, TI = 900 ms, flip angle = 9°, FOV = 240 mm, and 0.9 mm^3^ voxel resolution. For reference, a standard double echo gradient echo sequence was performed prior to the MREG-acquisition, which is used to calculate coil sensitivity profiles as well as a 3D fieldmap. Settings at 3T were: FOV = 192 mm: TR = 1000 ms, TE1 = 2.3 ms, TE2 = 4.6 ms, α = 50° and 64 slices (slice thickness = 3 mm). Cardio-respiratory frequencies were verified using physiological data recordings from the scanner, which were captured using an MR-compatible respiratory belt, cardiac quatrode, and integrated recording hardware during the scanning process.

Preprocessing was performed in SPM12, AFNI, and FSL following Nenert et al. (2025). Volumes were realigned for motion correction, with visual inspection for each participant, co-registered to each participant's T1-weighted MPRAGE, and normalized to MNI space (2x2x2mm). Realignment parameters were inspected for each participant and each run; all participants showed motion within accepted thresholds (below 3 mm translation and 3° rotation, corresponding to the ≤3 mm isotropic voxel dimension of the MREG acquisition). No participants or data frames (volumes) were excluded due to motion. MPRAGE scans were visually inspected for motion-related artifacts (ghosting, blurring), and no scans required exclusion. A 6 mm FWHM Gaussian kernel was applied for spatial smoothing, and signals were high-pass filtered at 0.008 Hz to remove slow drifts.

Physiological pulsations were quantified in two parallel analyses (spectral power and optical flow). For spectral power, AFNI's 3dPeriodogram computed voxelwise power in three bands—low-frequency (0.008–0.1 Hz), respiratory (0.11–0.44 Hz), and cardiovascular (0.52–1.6 Hz)—and the resulting maps from both runs were averaged per participant. For optical flow, a three-dimensional Lucas–Kanade algorithm (Pohl et al., 2021) estimated deformation vector fields between successive volumes; flow-magnitude time series were band-pass filtered into the same three bands and averaged across runs (Nenert et al., 2025).

Group differences between HC and PD were assessed in SPM12 by two-sample *t*-tests separately for power and flow within each band. Age and sex were entered as nuisance covariates. Statistical significance was determined at *p* < 0.05, cluster-level FDR-corrected. Anatomical labeling of significant clusters was performed using the Harvard-Oxford cortical atlas. Cohen's d effect sizes were computed from peak t-values and group sample sizes.

The data that support the findings of this study are available from the corresponding author with IRB approval upon request.

## 3 Results

### 3.1 Power Spectrum Analysis

In the cardiovascular spectral-power band, HC exhibited greater power than PD participants in six cortical regions: left middle orbitofrontal cortex, left fusiform gyrus, right inferior temporal cortex, left anterior cingulate, left middle frontal gyrus, and left cuneus (FigureTable). No significant group differences were observed in the low-frequency or respiratory spectral-power bands, and there were no regions where PD participants’ spectral power bands exceeded that of HC.

**Figure:**
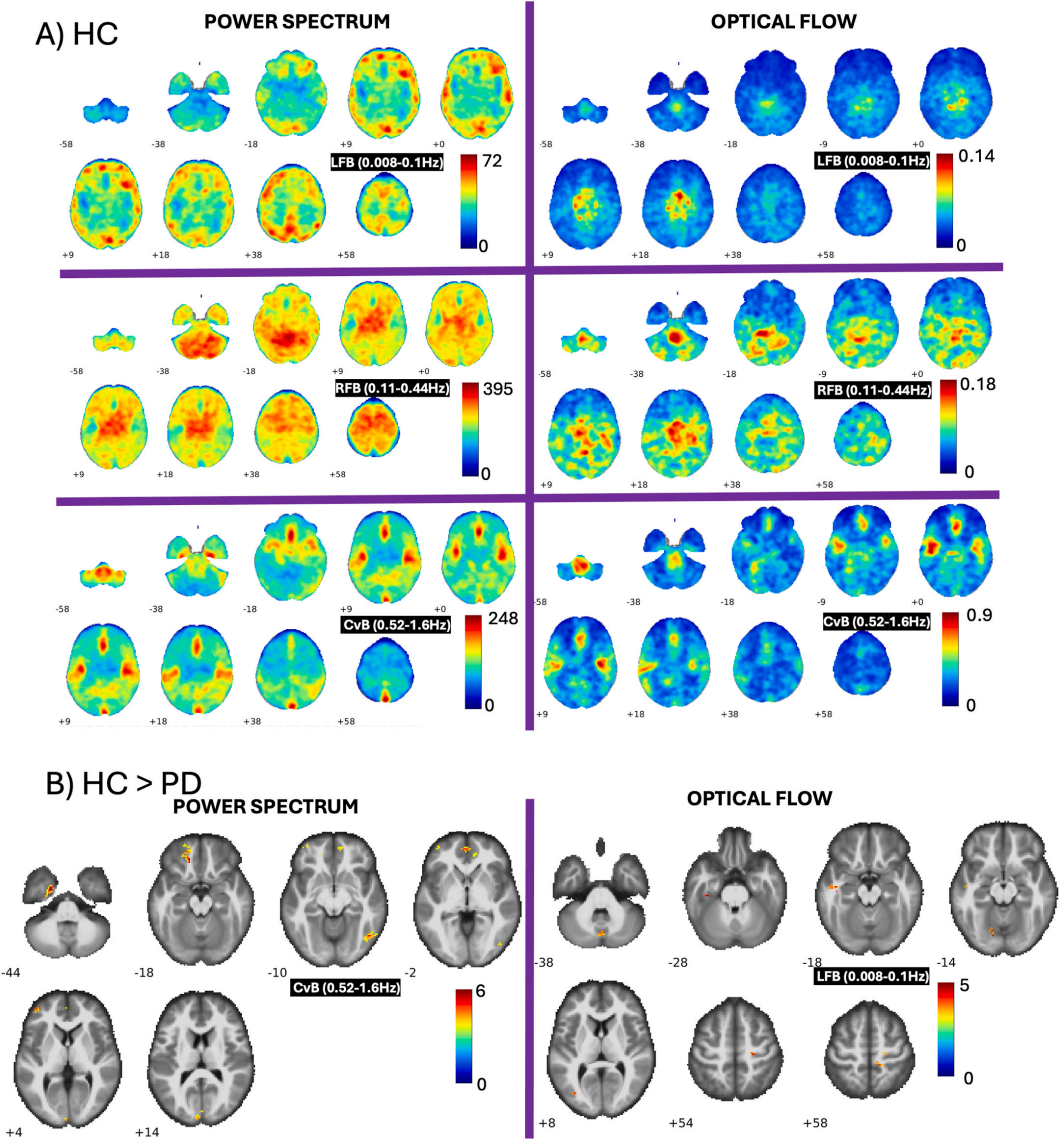
**Group differences in brain physiological pulsations (HC > PD)**. (A) **Cardiovascular frequency band spectral power** shows higher power in HC within orbitofrontal, fusiform, inferior temporal, anterior cingulate, middle frontal, and cuneus regions. (B) **Low-frequency/vasomotor frequency optical flow magnitude** is higher in HC within lingual, fusiform, middle temporal, middle occipital cortices, and the paracentral lobule. Maps are thresholded at **cluster-level FDR p < 0.05**. Color bars indicate *t*-values. Axial slices are displayed in neurological convention; coordinates are MNI.

**Table:** Brain regions showing significantly reduced physiological pulsations in Parkinson's disease compared to healthy controls.

Type	Freq band	Region Label	t-value	Cohen's d	MNI Coordinates		
					x	y	z
Power spectrum	CvB	Left middle Orbitofrontal	6.04	2.15	−16	38	−18
		Left Fusiform	5.89	2.10	−24	−4	−44
		Right inferior Temporal	5.22	1.86	48	−66	−10
		Left anterior cingulate	5.21	1.86	−4	52	−2
		Left Middle Frontal	4.74	1.69	−42	52	4
		Left Cuneus	4.58	1.63	−4	−96	14
Optical Flow	LB	Left Lingual	5.16	1.84	−14	−80	−14
		Left Fusiform	4.94	1.76	−38	−26	−28
		Left Middle Temporal	4.92	1.75	−46	−14	−18
		Left Middle Occipital	4.50	1.60	−36	−76	8
		Right paracentral lobule	4.26	1.52	14	−38	58

Regions are listed for cardiovascular spectral power and low frequency optical flow magnitude, including laterality, anatomical label, peak t-value, Cohen's d effect size, and Montreal Neurological Institute (MNI) coordinates (x, y, z). Cohen's d was computed from peak t-values using d = t × √(1/n_1_ + 1/n_2_), where n_1_ = 13 (PD) and n_2_ = 20 (HC). All differences indicate Healthy Control > Parkinson's disease (p < 0.05, cluster-level FDR-corrected, controlling for age and sex). Anatomical labeling was performed using the Harvard-Oxford cortical atlas. CvB: cardiovascular frequency band spectral power; LB: low frequency band optical flow magnitude.

### 3.2 Optical Flow Analysis

For optical flow magnitude in the low-frequency band, HC showed higher flow than PD in five regions: left lingual gyrus, left fusiform gyrus, left middle temporal gyrus, left middle occipital gyrus, and right paracentral lobule (Figure and Table). Optical flow in the respiratory and cardiovascular bands did not reveal any significant group differences, and no regions showed greater flow in the PD group.

### 3.3 Discussion

To our knowledge, this study is the first to use MREG to demonstrate that individuals with PD exhibit alterations in physiological pulsations thought to contribute to CSF dynamics and glymphatic flow compared to similarly aged healthy controls. The observed pattern of reduced cardiovascular-band power and low-frequency/vasomotor optical flow in PD suggests concurrent impairments in vascular pulsatility and slow-wave fluid dynamics as potential markers of PD-related neurodegenerative changes. Cardiovascular-band MREG fluctuations predominantly reflect cardiac-driven intracranial pulsations and neurovascular coupling. Their attenuation in orbitofrontal, fusiform, inferior temporal, anterior cingulate, middle frontal, and cuneus regions suggests impaired arterial compliance or weakened coupling between cardiac cycles and regional perfusion in PD, affecting areas important for visual and cognitive processing and decision-making. Such cerebrovascular dysfunction may compromise glymphatic clearance pathways and metabolic support, potentially exacerbating α-synuclein aggregation (Lopes et al., 2025).

Low-frequency optical flow indexes slow-varying tissue displacement linked to interstitial fluid movement along perivascular routes (Rajna et al., 2019). The observed diminution in occipito-temporal cortices (lingual, fusiform, middle temporal, middle occipital) and sensorimotor cortex (paracentral lobule) may indicate alteration in perivascular fluid transport or glial–vascular coordination in PD. This aligns with glymphatic-system models implicating perivascular channels in waste clearance (Iliff et al., 2012) and with reports of blood–brain barrier breakdown and neurovascular unit dysfunction in neurodegenerative disorders (Sweeney et al., 2018).

Notably, no significant group differences were observed in the low-frequency or respiratory spectral power bands, nor in respiratory or cardiovascular optical flow. There are several potential explanations. First, the small sample size may have limited statistical power to detect more subtle differences in these measures. Second, PD-related pathophysiology may differentially affect the mechanisms underlying each pulsation type and measurement approach. Spectral power and optical flow capture distinct aspects of physiological pulsations — power reflects the amplitude of signal fluctuations at a given frequency, while optical flow quantifies tissue displacement — and these may not be uniformly affected across all frequency bands. Third, respiratory pulsations are primarily driven by mechanical thoracic pressure changes, which may be relatively preserved in mild-to-moderate PD compared to cardiovascular and vasomotor mechanisms that depend more heavily on vascular integrity and neurovascular coupling. That said, respiration has been shown to be a major driver of human CSF flow in real-time MRI studies (Dreha-Kulaczewski et al., 2015) and modulating respiration alters glymphatic-lymphatic function in experimental models (Ozturk et al., 2023); the relative contribution of respiration compared to cardiac and vasomotor mechanisms to glymphatic flow remains an active area of investigation. Finally, the absence of regions showing greater pulsations in PD than HC suggests that the disorder is associated with a unidirectional reduction rather than a redistribution or compensatory increase in physiological pulsations, consistent with an overall decline in the pulsatile drivers of glymphatic flow.

Glymphatic clearance is known to decrease with aging and the current work using a proxy of glymphatic clearance shows that these alterations are further exacerbated in PD beyond what is seen in healthy older adults. This aligns with prior work in animal models and in humans showing changes in glymphatic clearance in PD (Lopes et al., 2025; Wood et al., 2024). Importantly, this reduction in glymphatic clearance has been associated with faster progression of both motor symptoms and cognitive decline in PD (Wood et al., 2024). Similarly, brain tissue pulsatility measured with resting state blood oxygenation level dependent (BOLD) MRI in white matter was found to be associated with motor symptoms in PD, although this fMRI technique did not have the high temporal resolution achievable by MREG to distinguish between frequency bands (Shirzadi et al., 2018). In the current work, alterations in cardiovascular frequency band and vasomotor flow in areas important for visual and cognitive processing support the importance of glymphatic flow in cognitive function in PD. Further, the paracentral lobule finding is consistent with functional-imaging evidence of early sensorimotor network alterations in PD, including impaired connectivity and network dynamics related to gait alterations(Shine et al., 2013). Together, these dual vascular and perivascular deficits may contribute to both motor and non-motor symptomatology by hindering nutrient delivery and waste removal.

Strengths of this work include the novel neuroimaging analysis with MREG as a marker of glymphatic flow and the case-control comparison. Limitations include the small sample size, which may introduce type II error, resulting in the inability to detect additional significant differences between groups. Nevertheless, this proof-of-concept study provides preliminary effect size estimates that can inform power calculations for future, adequately powered confirmatory studies. Additionally, while the reductions in cardiovascular band power were interpreted as representing altered neurovascular coupling, the observed reductions may also reflect disrupted peripheral cardiovascular function, which is common in PD and was not directly assessed in this study. Of note, an eyes-open paradigm without visual fixation was used in this study. Although this protocol was consistent across all participants, we acknowledge that fixation conditions could indirectly influence vigilance and arousal states, with potential downstream effects on physiological pulsation measures. The effect of fixation conditions on MREG-derived physiological pulsations has not, to our knowledge, been directly examined and warrants investigation in future studies. Finally, MREG in PD was not performed in the off-medication state and it is possible that levodopa or other medications may have influenced the results. Future work should evaluate relationships between these outcomes and behavioral measures in PD--including motor and non-motor symptoms--and longitudinally assess whether these MREG-derived markers predict clinical progression or respond to interventions targeting cerebrovascular health and glymphatic function.

In summary, this work is the first to demonstrate that brain physiological pulsations are altered in PD compared to similarly aged healthy older adults. The reduced cardiovascular-band power and low-frequency optical flow in PD, as indirect markers of pulsatile drivers of glymphatic flow, are consistent with the hypothesis that glymphatic dysfunction may contribute to PD pathophysiology and represent a potential therapeutic target aimed at altering the progression of neurodegenerative proteinopathies such as PD.

## Financial disclosures for the previous 12 months

Dr. Nenert has nothing to disclose.

Dr. Catiul has nothing to disclose.

Ms. Miften has nothing to disclose.

Dr. Levan receives research support from Canadian Institutes of Health Research (PJT-183825); Natural Sciences and Engineering Research Council of Canada (RGPIN-2021-02797); and Canada Foundation for Innovation (#42519).

Dr. Szaflarski receives funding from NIH, NSF, DoD, Charles Shor Foundation for Epilepsy Research, UCB Biosciences, NeuroPace Inc., SAGE Therapeutics Inc., Serina Therapeutics Inc., LivaNova Inc., Greenwich Biosciences Inc., Jazz Pharma, Biogen Inc., Eisai Inc., UniQure, State of AL; Consulting/Advisory Boards: PureTech Health, Biopharmaceutical Research Company, LivaNova Inc., UCB Pharma, AdCel Pharma, iFovea Inc.; Editor-in-Chief, Epilepsy & Behavior Reports (paid); Editorial board member for Epilepsy & Behavior, Journal of Epileptology (associate editor), Journal of Medical Science, and Folia Medica Copernicana; Dr. Szaflarski has served on the Alabama Medical Cannabis Commission (2021-2024; nominated by Dr. Scott Harris, State Health Officer).

Dr. Amara is a member of the faculty of the University of Colorado and is supported by University funds. She has served as a site investigator for studies sponsored by Michael J Fox Foundation for Parkinson's Research, Parkinson Study Group, Aligning Science Across Parkinson's (ASAP) Initiative, Koneksa Health, Biohaven Pharmaceuticals, Inc., Bial, and the NIH NINDS. She receives grant funding from NIH NICHD. She has received honoraria for consultancy to Aevum through Parkinson Study Group. She is a consultant for PhotoPharmics, Inc. and Aspen Neuroscience, Inc.

## Ethical compliance statement

We confirm that we have read the Journal's publishing ethics statement and affirm that this work is consistent with those guidelines. All participants provided written informed consent under UAB Institutional Review Board approval.

## Funding sources for the study

This study was funded by NIH NICHD R01HD100670 (AWA), the UAB Center for Exercise Medicine, the UAB Hypertension Research Center, and the UAB Evelyn F McKnight Brain Institute. MREG development was supported by the State of AL General Fund (“Carly's Law) and NIH/NINDS R01NS140278 (JPS).

## CRediT authorship contribution statement

**Rodolphe Nenert:** Conceptualization, Data curation, Formal analysis, Investigation, Methodology, Validation, Writing – original draft. **Corina Catiul:** Data curation, Investigation, Writing – review & editing. **Pierre LeVan:** Conceptualization, Methodology, Validation, Writing – review & editing. **Aya M. Miften:** Data curation, Investigation, Writing – review & editing. **Jerzy P. Szaflarski:** Conceptualization, Methodology, Supervision, Writing – review & editing. **Amy W. Amara:** Conceptualization, Funding acquisition, Investigation, Project administration, Supervision, Writing – original draft.

## Declaration of competing interest

The authors declare the following financial interests/personal relationships which may be considered as potential competing interests: Amy W Amara reports financial support was provided by National Institute of Child Health and Human Development. Amy W Amara reports financial support was provided by UAB Center for Exercise Medicine. Amy W Amara reports financial support was provided by The University of Alabama at Birmingham Evelyn F McKnight Brain Institute. Amy W Amara reports financial support was provided by UAB Hypertension Research Center. Jerzy P Szaflarski reports financial support was provided by National Institute of Neurological Disorders and Stroke. Dr. Nenert has nothing to disclose. Dr. Catiul has nothing to disclose. Ms. Miften has nothing to disclose. Dr. Levan receives research support from Canadian Institutes of Health Research (PJT-183825); Natural Sciences and Engineering Research Council of Canada (RGPIN-2021-02797); and Canada Foundation for Innovation (#42519) Dr. Szaflarski receives funding from NIH, NSF, DoD, Charles Shor Foundation for Epilepsy Research, UCB Biosciences, NeuroPace Inc., SAGE Therapeutics Inc., Serina Therapeutics Inc., LivaNova Inc., Greenwich Biosciences Inc., Jazz Pharma, Biogen Inc., Eisai Inc., UniQure, State of AL; Consulting/Advisory Boards: PureTech Health, Biopharmaceutical Research Company, LivaNova Inc., UCB Pharma, AdCel Pharma, iFovea Inc.; Editor-in-Chief, Epilepsy & Behavior Reports (paid); Editorial board member for Epilepsy & Behavior, Journal of Epileptology (associate editor), Journal of Medical Science, and Folia Medica Copernicana; Dr. Szaflarski has served on the Alabama Medical Cannabis Commission (2021-2024; nominated by Dr. Scott Harris, State Health Officer). Dr. Amara is a member of the faculty of the University of Colorado and is supported by University funds. She has served as a site investigator for studies sponsored by Michael J Fox Foundation for Parkinson's Research, Parkinson Study Group, Aligning Science Across Parkinson's (ASAP) Initiative, Koneksa Health, Biohaven Pharmaceuticals, Inc., Bial, and the NIH NINDS. She receives grant funding from NIH NICHD. She has received honoraria for consultancy to Aevum through Parkinson Study Group. She is a consultant for PhotoPharmics, Inc. and Aspen Neuroscience, Inc.

If there are other authors, they declare that they have no known competing financial interests or personal relationships that could have appeared to influence the work reported in this paper.

## Data Availability

Data will be made available on request.
